# Transplantation of Teeth

**Published:** 1896-05

**Authors:** 


### TRANSPLANTATION OF TEETH.

Dr. E. S. Pettyjohn, superintendent of the Alma Sanitarium, reports in the mid-January number of the *Medical Fortnightly* an interesting and successful case of teeth transplantation. The patient, his little daughter, aged two years and nine months, fell headlong down the cellar stairs, completely extracting, as if by forceps, the two upper middle incisors on the edge of the step. The gum was lacerated considerably. The teeth were placed in a tepid normal saline solution, and about an hour after the accident were replaced under chloroform and pressed into position with the aid of forceps. The adjacent gums were rendered aseptic, but no further dressing or application was made. The child awoke in two hours and a half. Milk and soft food were administered, cleans-ng the gums after eating. Healing took place by first in-e ntion. At the time of writing, four weeks after the acci dent, the teeth were in position and of normal appearance, as was also the gum.

---

THE following interesting case is reported by Dr. John A. Wyeth, in the *Nashville Journal of Medicine and Surgery*:

"Henry Ellman, of Providence, Rhode Island, 20 years of age, entered my service in the New York Polyclinic Hospital July 3rd, 1895, with a history of having been operated upon at his home for appendicitis three months previous. There was a fæcal fistula extending from the colon through the line of incision of the operating wound. Prolonged effort was made to close up this fistula by the opening up of the sinuses communicating with it and by careful diet which would leave the minimum of undigested matter. As he was not cured by September 29th, I made an explatory incision and found that the cæsum was involved, there being a wide opening in the outer portion of this intestine, and no operation promised success without exsection of the gut. As the patient was in no condition to justify such a formidable procedure, I decided to attempt a lateral anastosis between the descending colon and the ileum, in order to divert the intestinal current away from the cæcum. On October 1, I did a rapid lateral anastomosis as above by the aid of a middle-sized Murphy button. The patient re-acted well, gained rapidly in health and strength for four weeks following the operation. The discharge from the fistula decreased and the movements by rectum became almost normal.

The latter part of November, the fæcal discharge from the fistula suddenly increased again and the patient began to lose in flesh. He was discharged December 12, in about the same condition as before the operation, still retaining the Murphy button, which caused him comparatively little discomfort. He returned to Providence, and on Tuesday, February 4, 1896, one hundred and twenty-seven days after the operation, Dr. Whitemarsh, of that city, removed the button."

The Doctor writes: "I found the button in the ileum about three feet from the point of anastomosis, it having traveled in the direction of the ileo-cæcal valve. The colon was still contracted in spite of one or two movements a day per rectum. I could not slide the button along into the colon through the gut at the fistula. I therefore made a slight longitudinal incision, squeezed the button through, and stitched up the opening made. There was no rise of temperature after the operation, and the patient is doing fairly well."

